# The Intra-Dependence of Viruses and the Holobiont

**DOI:** 10.3389/fimmu.2017.01501

**Published:** 2017-11-09

**Authors:** Juris A. Grasis

**Affiliations:** ^1^Department of Biology, San Diego State University, San Diego, CA, United States; ^2^School of Natural Sciences, University of California at Merced, Merced, CA, United States

**Keywords:** holobiont, virome, symbiosis, viral metagenomics, host–microbe interactions, innate immunity, antiviral immunity, bacteriophage

## Abstract

Animals live in symbiosis with the microorganisms surrounding them. This symbiosis is necessary for animal health, as a symbiotic breakdown can lead to a disease state. The functional symbiosis between the host, and associated prokaryotes, eukaryotes, and viruses in the context of an environment is the holobiont. Deciphering these holobiont associations has proven to be both difficult and controversial. In particular, holobiont association with viruses has been of debate even though these interactions have been occurring since cellular life began. The controversy stems from the idea that all viruses are parasitic, yet their associations can also be beneficial. To determine viral involvement within the holobiont, it is necessary to identify and elucidate the function of viral populations in symbiosis with the host. Viral metagenome analyses identify the communities of eukaryotic and prokaryotic viruses that functionally associate within a holobiont. Similarly, analyses of the host in response to viral presence determine how these interactions are maintained. Combined analyses reveal how viruses interact within the holobiont and how viral symbiotic cooperation occurs. To understand how the holobiont serves as a functional unit, one must consider viruses as an integral part of disease, development, and evolution.

## Introduction

All animals interact with a consortium of microbes at all times and have done so since the dawn of animal life (1). Animal life has evolved from and in intimate association with microorganisms, while these same microorganisms have evolved in part to the resources provided by their animal surroundings. This symbiosis allows for a sharing of resources, including metabolic products and genes. These interactions have been of intense research and speculation; however, an important player in these symbiotic interactions is often overlooked, the effects of viruses. None of these interactions occur in the absence of viruses, so to inquire about symbioses requires discussion of viruses.

Viruses are seemingly universal in the biosphere (2). Their numbers are so staggering that when speaking of large numbers, one should use the term “viral” rather than “astronomical.” There are an estimated 10^31^ viruses on the planet, which may be an underestimation due to our inability to properly enumerate RNA viruses and viral elements that persist in cells and genomes (3). Further, viral genomes are worldwide reservoirs of genetic diversity (4). Considering viral abundances, diversity, and ubiquitous presence (5), understanding symbioses is lacking without taking into account the effects of viruses on host and associated microbe metabolism, and genetic flow between organisms.

Viruses infect all animals, from Poriferans to Cnidarians to Bilaterans to Chordates. There is ever-increasing evidence that viral infections have occurred during all of cellular life, as the presence of viral elements are often found in genomes throughout evolution (6). Host–viral infections or associations are not adequately quantified, but in most host-associated systems it seems that the number of viruses is equivalent to or slightly less than the number of bacteria associating with a eukaryotic host (2, 7). In most cases, the enumerable viral populations are the free DNA prokaryotic viruses, which are likely involved with the regulation of the host-associated bacteria. In host-associated systems, it seems that Lotka–Volterra “kill-the-winner” predator–prey dynamics of the prokaryotic virus and bacteria are atypical. Many prokaryotic viruses found in these systems display temperate lifestyles in which the virus becomes latent and integrates into a host chromosome or exists as an episomal element, as indicated by the large abundance of integrase genes in viral genomes (8, 9). Additionally, the presence of latent viruses may allow for bacterial dominance of a niche in the presence of related strains (10). Experimental evidence in non-host-associated systems supports this idea, as increasing concentrations of bacteria favor prokaryotic virus temperate lifestyles (11). While most viral research focuses on lytic/virulent infections, it is useful to explore both the temperate dynamics of prokaryotic viruses and latent eukaryotic viral infection, and their role in symbiosis.

The functional association between a host, prokaryotic, eukaryotic, and viral entities within a particular environment is the holobiont. This functional association helps to define the phenotypic unit. Casual associations may not define the phenotype, so functional associations (and the genes used) help define the phenotype. This functional symbiosis is involved in animal development (12), nervous system regulation (13, 14), immune system development and regulation (15, 16), and many other biological processes (17). When this functional association breaks down, a dysbiotic state occurs, leading to grave effects on animal health, ranging from coral bleaching (18), to stunted immune system development (19), to nervous and immunological disorders (20), to effects on human health (21). Further, the holobiont is not static; it is in a constant state of genetic flux. Viruses predominately affect this genetic flow and the acquisition of evolutionary traits (22, 23). Therefore, understanding the holobiont requires investigation of the effects viruses have on gene flow occurring within it. This is evaluated through viral metagenomics (viromics), where culture-independent viral isolations from host systems are sequenced and the viral genomes are analyzed. Not only can host-associated viral populations be identified, but how these populations change under dysbiotic conditions (24, 25), the identification of new viruses (26, 27), and the effects these viruses have on cellular systems (28) can all be learned through viral metagenome (virome) analyses.

## Viral Symbioses as Parasitism

Viruses act as parasites; they infect and either replicate within the host cell or integrate within the host genome. Viruses propagate by one of two different lifestyles, either lytic/virulent or temperate/latent. The lytic/virulent lifestyle involves the infection, replication, and lysis of the cell, leading to the death of the cell and release of viral progeny. The temperate/latent lifestyle involves the integration of the virus into the genome in a proviral form, which can be activated at a later time to become a lytic/virulent replicative virus. Either one of these scenarios affects the host; replication leads to cellular damage, while integration leads to genomic damage. The host defense against parasitism limits cellular or genomic damage (29). These viral parasitic lifestyles cause a molecular arms race, the virus seeking a new host to continue propagation, while the host immune system recognizes the virus to minimize damage (30).

There are many direct causes of pathogenesis by parasitic viruses, but there are many indirect causes as well. Proviral endogenous retroelements can have negative effects on the genome by inserting, deleting, or rearranging portions of the genome (31). The large number of freely associating viruses found interacting with host systems also presents a conundrum, that the presence of large amounts of viral material, be it nucleic acid or protein, makes it unlikely that they would not cause an immune response. Microbial-associated molecular patterns (MAMPs) on prokaryotic and eukaryotic viruses can cause immune system recognition that can lead to immune related pathogenesis. Further, lysis of cells, be it of a bacterial cell or of a eukaryotic cell, or apoptosis of a virally infected cell can cause activation of the immune system leading to pathogenesis (32). Cellular lysis is often considered in the aftermath of eukaryotic viral infection, but prokaryotic lysis of bacteria is commonly overlooked. Release of bacterial antigens, such as LPS, peptidoglycans, lipopeptides, lipoteichoic acid, flagellin, and bacterial DNA, can easily activate the immune system, and in extreme cases lead to sepsis (33). There are many direct and indirect causes of viral pathogenesis, but given the sheer numbers of viruses within a holobiont, and the limited pathogenesis that actually occurs, it seems more likely that viral pathogenesis is not as common as viral commensalism and mutualism.

## Viral Symbioses as Commensalism and Mutualism

Most consider viruses to be parasites, where infection benefits the virus, but decreases the fitness of the host. Now consider other scenarios, such as commensals and mutualists. A virus can be commensal, the virus benefits while host fitness is unaffected. A virus can be mutualistic, in which both organisms benefit and fitness increases. Such viral associations may provide advantages that promote evolution and biodiversity (34, 35). Also consider that one virulent virus among a sea of non-virulent viruses does not equate to pathogenesis. Unless transmission and recovery rates are high, pathogenicity may be an evolutionarily poor strategy for viral survival. More likely, pathogenesis is the exception and not the rule, with more instances being discovered of viruses having cooperative roles with the host (34, 36).

There are many instances where an organism cannot exist without beneficial viruses. Polydnavirus integration into parasitoid wasp genomes counters the effects of the caterpillar host immune system where the wasp has laid its eggs (37). Without this polydnavirus presence, the caterpillar immune system would eliminate the wasp eggs, but when the polydnavirus endogenous viral element becomes active upon egg deposition, the host immune response to the eggs is negated. Similarly, endogenous retrovirus syncytin expression in the placenta of mammals allows for the development of the placental syncytium (38). This syncytial fusion creates a barrier for the placenta, which in part keeps the fetus from being rejected by the mother’s immune system. Viruses can also modulate the immune system and restore dysbiotic conditions. Kernbauer et al. have shown that an enteric murine norovirus can restore normal mucosal immunity and intestinal morphology in germ-free mice, essentially replacing the immune stimulatory effects of gut microbiota (39). Viruses can also protect against or impede further infection or pathogenesis, such as Hepatitis G virus slowing the progress of HIV infection (40), and latent herpesviruses protecting against bacterial infections (41). It is becoming evident that viruses have the potential to be something more than parasites in a holobiont, which revises conceptions of how viruses impact host interactions.

## I am One with the Viruses, the Viruses are with Me

Viruses can also integrate into cellular genomes and act as genetic elements associating with genomes. The amount of DNA of viral origin within the human genome is similar to that of human coding domains (42). One major discovery in viromes is the persistence of viral genetic elements, either latently integrated into host genomes or surviving as chromosomal episomes. Host-associated viral populations seem to be dominated by temperate prokaryotic viruses or latent eukaryotic viruses. This is attributed to a large abundance of integrase sequences in prokaryotic viromes (8) and a large abundance of transposase sequences in eukaryotic viromes (43).

Integrated viral DNA in the host genome are endogenous viral elements (EVEs), which have the potential to drive evolutionary processes, such as speciation, resulting in the emergence of new traits (44–46). In addition to these evolutionary transitions, EVE integration can affect gene expression through their long terminal repeats (LTRs). These LTRs are repetitive viral DNA sequences that flank integrated EVEs, serving as promoters to both viral and host genes. These LTRs can affect stem cells (47), development (48), and immunity (49, 50). There are many individual genes affected by EVEs, though their major impact on evolutionary traits may be on gene regulatory networks, or the cellular regulators that impact RNA and protein expression (51, 52). The effects of EVEs and transposable elements in all these biological processes are being recognized as vitally important (53).

Genomically integrated viral elements are reminders that viruses affect everything in biology, but what about free viruses that associate with hosts? Viromics allow researchers to analyze the viral populations and effects these viruses have on the holobiont. These studies have been conducted in many host systems, from the base of animal life in the Cnidarian phylum (54) to mammals (55). Often, the viruses found freely associating are prokaryotic viruses, which regulate the number and strains of bacteria in a holobiont (56). These viruses are likely selected by the host to maintain bacterial populations (26). Further, viromics show the sphere of viral involvement in gene flow and gene shuffling in an ever-changing environment, often from within bacterial cells and sometimes from within eukaryotic cells.

## The Eternal Struggle of Host–Viral Interactions

**Figure 1:**
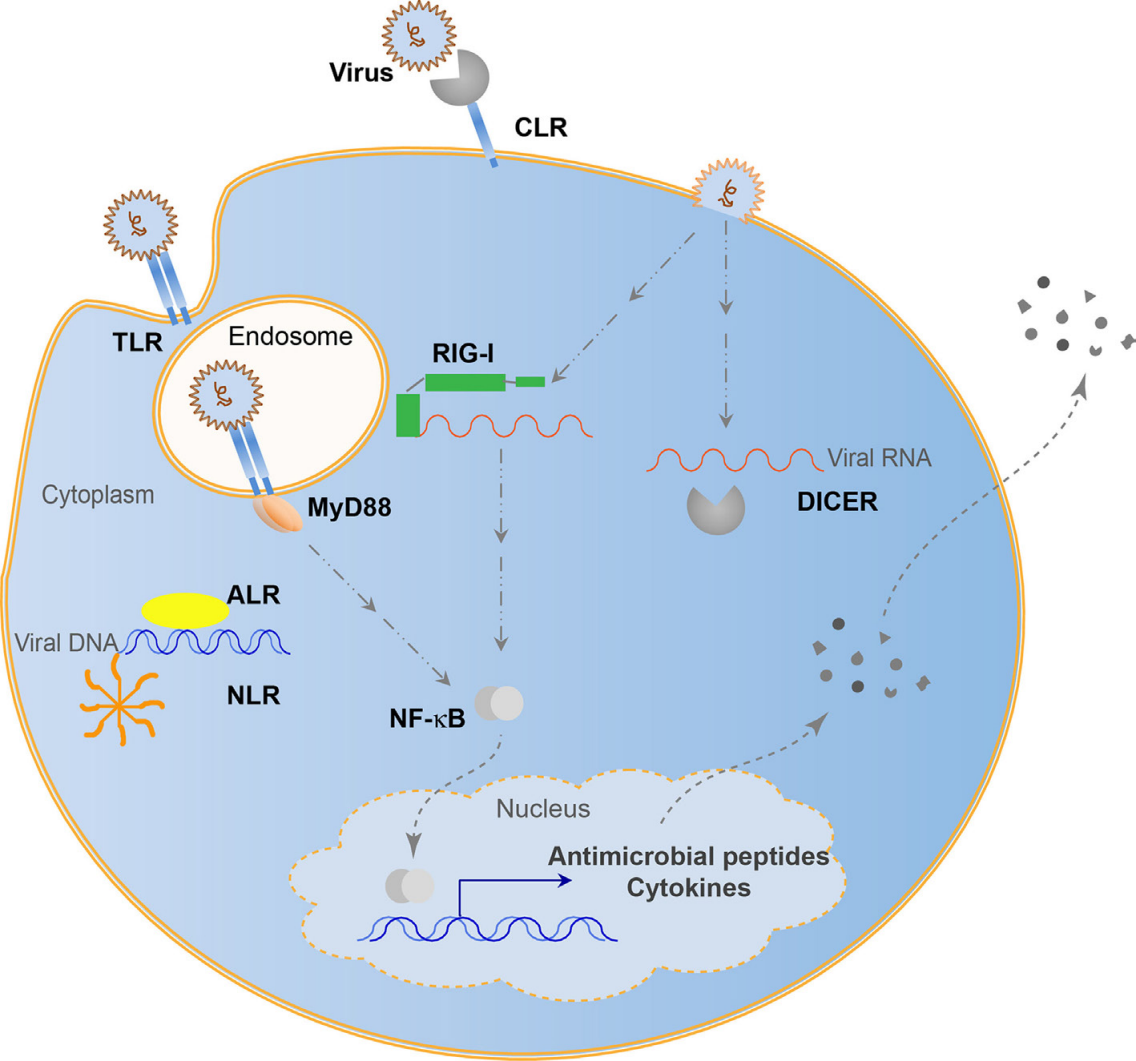
Evolutionarily conserved antiviral innate immune systems. Toll-like receptors (TLRs) recognize endosomal viral nucleic acids, NOD-like receptors (NLRs) form an inflammasome and recognize viral DNA, absent-in-melanoma-like receptors (ALRs) recognize viral DNA, retinoic acid-inducible gene I (RIG-I) and RNAi (Dicer) pathways recognize viral RNA, while C-type lectins (CLRs) recognize viral carbohydrates. Some pathways can lead to the direct elimination of viral entities, while others lead to transcriptional activation resulting in cytokine and antimicrobial peptide secretion.

Many viruses can persist in host cells and influence the host without symptoms of disease. Chronic systemic viruses continuously stimulate the immune system (57), driving the emergence of many viral recognition systems over evolutionary time (58). These recognition systems give a host integrity to coexist with viruses while minimizing pathogenesis and protecting genomic information. Antisense RNA encoded by genomic transposable elements allows for specific regulation of viral amplification products (59). This evolved into use of antisense RNAs with Argonaute nucleases. Piwi-interacting RNAs utilize transposon derived small RNAs to defend against integration events by binding to complementary RNAs and cleaving the complex with a bound Argonaute nuclease. This system seems to be restricted to the germ-line and protects genomic integrity. Similarly, the RNAi system processes RNAs by binding to small RNA fragments and cleaving these complexes with an RNase III nuclease, Dicer (60). While controversial, it appears that chordates may not have retained RNAi antiviral function. However, there are many immune functions additionally used in both chordates and non-chordates to regulate viral presence (Figure 1). These systems rely on host pattern-recognition receptors (PRRs) evolved to recognize MAMPs. These PRRs include the Toll-like receptors (TLRs), retinoic acid-inducible gene I (RIG-I)-like receptors (RLRs), cGAS-STING pathway, NOD-like receptors (NLRs), C-type lectin receptors (CLRs), and absent-in-melanoma-like receptors (ALRs). TLRs recognize viruses endosomally once viral nucleic acids are released (61), cytoplasmic RLRs recognize viral genomic RNA or double-stranded RNA intermediates (62), cGAS-STING senses retroviral and double-stranded DNA (63), NLRs recognize viral DNA genomes (64), ALRs can also recognize viral genomic DNA (65), while CLRs recognize carbohydrates (66). In the biological arms race that caused the development of the adaptive immune system capable of tracking evolutionary changes in pathogens, antiviral cytokines such as interferons (IFNs) became prominent signals alerting the host of viral infection and inhibit viral propagation (67). With IFNs came recombination events to generate antibodies and major histocompatability complexes in vertebrates to increase the recognition possibilities that came with increased pathogen complexity. Although viral recognition research is often focused on the adaptive immune system in mammals, the overwhelming majority of animals has multiple pathways to recognize, regulate, and maintain viral associations and may not necessarily use canonical adaptive systems to structure the holobiont. Continuing research will involve the 95% of Metazoans that do not possess such an adaptive immune system to recognize viruses, yet are able to adapt to ever-changing viral populations through mechanisms, such as trained innate immunity (68).

## Hail *Hydra*: The Importance of a Simple Model System to Evaluate Holobiont Interactions

Holobiont studies are complex. If one considers the sheer number of associated prokaryotes, eukaryotes, viruses, and all of their respective genomes, the number of potential interactions is overwhelming. Therefore, if one can use a model system with a limited number of microbial partners to deconstruct the holobiont and if this can be studied in an ancient animal phylum for conserved holobiont interactions, it could simplify these studies while retaining informative and predictive capabilities. The use of a basal metazoan allows research on mechanisms of holobiont assembly, holobiont effects on microbiota and host health, and metabolic interactions between the host and microbiota. This helps to elucidate symbiosis in healthy states and dysbiosis in disease states.

**Figure 2:**
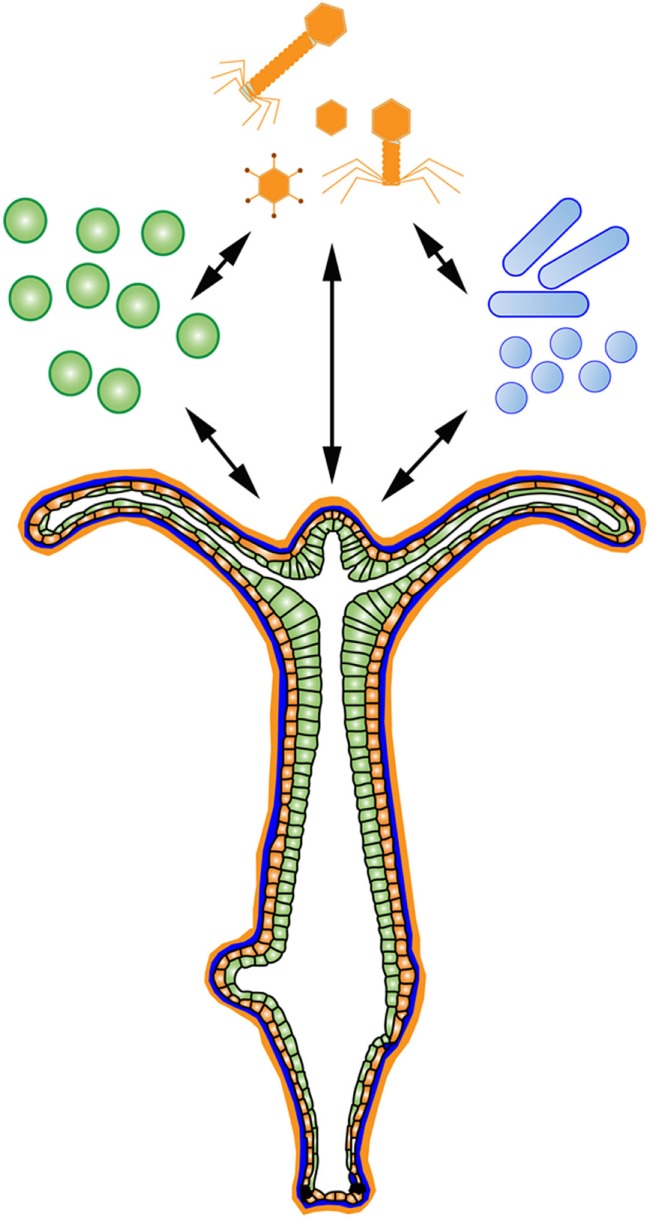
The *Hydra* Holobiont. *Hydra* are an ideal system to deconstruct and reconstruct an organismal holobiont consisting of associated eukaryotes (green), prokaryotes (blue), and viruses (orange) at an exposed epithelium.

There are many useful systems that meet the above criteria to investigate the holobiont, including ascidians (69), anemones (70), and sponges (71). The basal model organism *Hydra* is another useful system. *Hydra* are freshwater Cnidarians practical for developmental, neural, aging, and stem cell studies (72). Importantly, the findings made using *Hydra* translate well into host–microbe interaction studies due to its diploblastic morphology (73), conserved mucosal immunity (74), and limited number of microbial partners (75). Additionally, *Hydra* are clonal, have a well-annotated genome (76), can be made transgenic (77), germ-free (78), and due to its limited number of microbial interactions, *Hydra* can be used in symbiosis studies (79). *Hydra* display distinct microbial colonization patterns dependent on host factors (78), which are primarily driven by antimicrobial peptide selection at the epithelium (80). *Hydra* have many evolutionarily conserved receptor pathways to regulate microbial interactions, including a TLR pathway (81) and a large repertoire of NLRs (82). Further, *Hydra* utilize many uniquely identified classes of antimicrobial peptides to regulate its microbial interactions (81, 83, 84). Finally, 57% of the *Hydra* genome are transposable elements, one of the largest percentages found in an animal genome (76). These factors make *Hydra* a useful system to deconstruct and reconstruct an organismal holobiont (Figure 2).

Understanding the complete *Hydra*-associated virome has commenced. The *Hydra* DNA virome consists primarily of prokaryotic viruses in the Caudovirales order, the majority of the eukaryotic viruses are of the Herpesviridae family, the diversity of the viruses increases upon environmental heat stress, and each species of *Hydra* associates with a specific community of viruses (25). Further, these *Hydra*-associated viruses affect *Hydra*-microbiome metabolism (25, 85). Studies on the RNA virome, germ-free eukaryotic virome, and prokaryotic virome of *Hydra*-associated bacteria are ongoing to create a comprehensive *Hydra* virome [J. Grasis, in preparation; (86)]. Combining the virome with *in vivo* viral infection transcriptomes and the ability to induce inflammatory conditions makes *Hydra* a useful system to structure viral–holobiont interactions related to animal health conditions. The *Hydra* model system may shed light on novel aspects of holobiont formation, maintenance, and dysbiosis, while integrating viral involvement within the holobiont.

## Viruses Bring Balance to the Holobiont

There has been much discussion about the holobiont recently, particularly as it relates to selective units of animal host and microbiome (87–93). Much of the focus has been placed on host–bacterial associations, but what of the viruses? They are intrinsically part of the genome and part of the holobiont, and yet, extrinsically exist beyond the genome and the holobiont. This duality exists because both the host and the microbiome are under their own selective pressures, each are selecting for the environment that benefits them, establishing or propagating a phenotype, and allowing for co-existence to continue. It is neither eukaryo-centric, prokaryo-centric, nor viro-centric, each member has a role to play within the holobiont. Therefore, the holobiont is a coordination of integrated functions by all members to suit adaptation to an environment.

Viruses are genetic parasites constantly sampling their environments. Functional aspects of their genomes can be selected by their prokaryotic and eukaryotic hosts, and in this way, viruses are symbionts to these hosts. Viruses can also transfer DNA in the form of lateral gene transfer, which can be important for adaptations to new environments (94). For example, prophages can promote genetic transfer between prokaryotic viruses and eukaryotes. *Wolbachia* prophage WO in arthropods contains a eukaryotic association module, which among other genes, contains a spider toxin gene that can form pores in both prokaryotic and eukaryotic membranes to facilitate viral escape (95). There are many more instances of viral drivers of adaptation (96), which makes viral dynamics in the holobiont fluid. Free viruses can be acquired from the environment through horizontal transmission, while viral elements can be vertically transmitted through genomically integrated viral elements and episomes. Such horizontal and vertical transmissions allow for a fully functional range of symbioses, from obligate (both need each other to survive) to facultative (both benefit from the association, but it is not absolute).

Viruses are remarkable symbionts. Viral elements exist intra-genomically, intra-cellularly, extra-cellularly, and environmentally. They persist in all of these realms, and yet, are vital to the holobiont. As mentioned earlier, viromics teaches us that viruses are involved in gene flow and shuffling in a changing environment, and that the elements in the holobiont are in a constant ecological flux. In all cases, viruses provide balance to the holobiont, keeping the host and associating prokaryotes and eukaryotes functioning together as a unit.

## Author Contributions

JG wrote, did the artwork, and is responsible for the content of this manuscript.

## Conflict of Interest Statement

The author declares that the research was conducted in the absence of any commercial or financial relationships that could be construed as a potential conflict of interest.
